# QoL, CIs, QALYs, and Individualized Rehabilitation: The Clinical and Practical Benefits of Regularly Assessing the Quality of Life of Adult Cochlear Implant Recipients

**DOI:** 10.3390/ijerph20206906

**Published:** 2023-10-10

**Authors:** Luis Lassaletta, Miryam Calvino, Isabel Sanchez-Cuadrado, Piotr Henryk Skarzynski, Katarzyna B. Cywka, Natalia Czajka, Justyna Kutyba, Dayse Tavora-Vieira, Paul Van de Heyning, Griet Mertens, Hinrich Staecker, Bryan Humphrey, Mario Zernotti, Maximo Zernotti, Astrid Magele, Marlene Ploder, Julia Speranza Zabeu

**Affiliations:** 1 Department of Otorhinolaryngology, Hospital La Paz. IdiPAZ Research Institute, 28046 Madrid, Spain; miryamcf@yahoo.com (M.C.); iscuadrado@gmail.com (I.S.-C.); 2 Biomedical Research Networking Centre on Rare Diseases (CIBERER), Institute of Health Carlos III, (CIBERER-U761), 28029 Madrid, Spain; 3 Institute of Physiology and Pathology of Hearing, World Hearing Center, 05-830 Kajetany, Poland; p.skarzynski@ifps.org.pl (P.H.S.); k.cywka@ifps.org.pl (K.B.C.); n.czajka@ifps.org.pl (N.C.); j.kutyba@ifps.org.pl (J.K.); 4 Fiona Stanley Fremantle Hospitals Group, Perth 6150, Australia; dayse.tavora@gmail.com; 5 Department of Otorhinolaryngology, Antwerp University Hospital, 2650 Antwerp, Belgium; paul@vandeheyning.com (P.V.d.H.); griet.mertens@uza.be (G.M.); 6 ENT Department, University of Kansas Medical Centre, Kansas City, KS 66160, USA; hstaecker@kumc.edu (H.S.); bhumphrey@kumc.edu (B.H.); 7 Department of Otorhinolaryngology, Sanatorio Allende de Córdoba, Córdoba 5000, Argentina; mario.zernotti@gmail.com (M.Z.); maxi.zernotti@gmail.com (M.Z.); 8 ENT Department, Universitätsklinikum St. Pölten, 3100 St. Pölten, Austria; astrid.magele@stpoelten.lknoe.at (A.M.);; 9 Hospital de Reabilitacão de Anomalias Craniofaciais da Universidade de Sao Paulo, Campus Bauru, Bauru 17012-230, Brazil; julia.zabeu@usp.br

**Keywords:** cochlear implant, quality of life, rehabilitation, patient reported outcome measure, single-side deafness, asymmetrical hearing loss

## Abstract

This study aimed to report quality of life (QoL) scores in unilateral cochlear implant (CI) users and to generate guidance for clinicians on using QoL measures to individualize CI counselling and rehabilitation and to increase access to CIs as a mode of rehabilitation. Participants (n = 101) were unilateral CI users with single-sided deafness (SSD; n = 17), asymmetrical hearing loss (AHL; n = 26), or bilateral hearing loss (Uni; n = 58). Generic QoL was assessed via the Health Utilities Index (HUI-3), and disease-specific QoL was assessed via the Speech, Spatial, and Qualities of Hearing scale (SSQ12) and Nijmegen CI Questionnaire (NCIQ) at preimplantation and at 6 and 12 months of CI use. All groups had significantly increased HUI-3 scores at both intervals. The SSD group showed significant benefit on the SSQ12 at visit 3, the AHL group showed significant benefit on the SSQ12 and most NCIQ subdomains at both intervals, and the Uni group showed significant benefit with both tests at both intervals. Unilateral CI recipients demonstrate improved QoL within the first 12 months of device use. Regular assessment with generic and disease-specific questionnaires has the potential to play an important role in personalizing treatment and possibly in increasing access to CI provision.

## 1. Introduction

Cochlear implant (CI) use can provide a host of benefits, including a significantly improved health-related quality of life (HRQoL) [1,2,3,4]. As part of a trend towards viewing the effects of CI use holistically, participants’ self-assessed HRQoL is now more frequently used as an assessment measure in studies. HRQoL assessments are generally questionnaires or surveys and can be generic or disease specific. Generic measures, such as the Health Utilities Index (HUI-3) [5] or the Assessment of Quality of Life (AQoL)-8D [6], can be used to compare the effects of different interventions on HRQoL, e.g., benefit derived from CI use vs. derived from receiving a partial nephrectomy [7]. Disease-specific assessments appear to be more sensitive to changes in HRQoL after hearing implant provision than generic assessments; thus, they are an excellent tool to assess or monitor a candidate’s or user’s HRQoL [8,9]. Numerous disease-specific tests exist for hearing loss in general, e.g., the Speech, Spatial, and Qualities of Hearing scale (SSQ12) [10]; for specific device types, e.g., the Nijmegen Cochlear Implant Questionnaire (NCIQ) [11] or the Abbreviated Profile of Hearing Aid Benefit (APHAB) [12]; and for specific indications, e.g., single-sided deafness (SSD) [13] or bimodal users [14].

Having CI candidates and users self-assess their HRQoL with these generic- and disease-specific tools has several benefits. Firstly, they are, by definition, user-centric, so they assess the real-life effects of CI use. Secondly, regular pre- and postoperative assessments with disease-specific HRQoL measures could also allow the therapist to gauge the user’s satisfaction over time. Based on the answers, further rehabilitation sessions could be tailored to focus on situations that a user finds meaningful and challenging, thereby facilitating personalized therapy. Moving beyond the level of the individual CI candidate/user, generic assessments, unlike disease-specific assessments, can generate quality-adjusted life years (QALYs). This is important because QALYs are essential for creating the cost-benefit assessments that inform hearing healthcare policy. Thus, the results of generic assessments should enable more people to benefit from access to hearing devices because while the clinical benefits of intervention on many conditions are well-established, the cost-effectiveness is sometimes not [e.g., [7]].

To explore and quantify the usefulness of generic and disease-specific assessments, the aims of the present study were to (1) investigate changes in HRQoL after unilateral CI treatment across different indications in a multi-centric/multinational set-up and (2) propose how HRQoL measures could be used to individualize CI counselling and rehabilitation.

## 2. Materials and Methods

### 2.1. Participants

The participants in the present study were all also included in Lassaletta et al. [3]. In short, the inclusion and exclusion criteria were that they had to have postlingual hearing loss, have never used a hearing implant on the to-be-implanted side, be at least 10 years old, and give their informed consent.

A point of deviation from the aforementioned study is that the present study focuses only on CI treatment and rehabilitation; as such, users of middle ear implants (the VIBRANT SOUNDBRIDGE group) and bone conduction implants (the BONEBRIDGE group) were excluded. Moreover, out of the 111 original CI users, the electric-acoustic stimulation users (corresponding to the SONNET EAS audio processor group, n = 10) had to be excluded due to having an insufficient *n* for meaningful inferential statistics to be performed. Therefore, all of the remaining 101 participants in the present study were unilateral CI users with SSD, asymmetric hearing loss (AHL), or bilateral severe-to-profound deafness (herein called “unilateral”). SSD was defined as having a pure tone average of ≥70 dB HL in the poorer ear and ≤30 dB HL in the better ear. AHL defined as having a pure tone average of ≥70 dB HL in the poorer ear and between >30 and ≤55 dB HL in the better ear. Pure tone average is defined as the mean threshold at pure-tone frequencies of 0.5, 1, 2, and 4 kHz.

Prior to surgery, all candidates were given a thorough audiological workup. Radiological examinations, including temporal bone computerized tomography and magnetic resonance imaging, were routinely performed. The decision of whether to proceed with implantation was made after consultation with a multidisciplinary team. Surgery was performed by an experienced surgical team. The CIs were activated (i.e., first fitting) within the first month post-surgery, whereafter recipients began rehabilitation with speech language professionals.

### 2.2. Assessments and Intervals

The three study intervals were at pre-activation, at 6 months of device use, and at 12 months of device use. Participants completed the questionnaires via paper and pencil.

#### 2.2.1. Generic QoL

Generic QoL was assessed in all participants via the HUI-3 at all three intervals [5]. Scores on the HUI-3 range between 0.00 (i.e., dead) and 1.00 (i.e., in perfect health). Negative scores represent states that are considered worse than being dead. Mean differences of 0.03 are considered clinically important, and smaller differences, such as 0.01, can also be meaningful and important in some contexts [5]. To calculate the HUI-3 scores, all questions had to be answered by the study participant. Participants with missing items were not included in the analysis.

#### 2.2.2. Disease-Specific QoL

Disease-specific QoL was assessed at all three intervals via the SSQ12 [10] and the NCIQ [11].

The SSQ12 consists of 12 items with a visual analogue scale between 0–10, in which higher scores indicate better HRQoL. It was used to measure hearing abilities across three subdomains: speech perception, spatial hearing, and general qualities of hearing. In this study, we analyzed only the total score.

The NCIQ consists of three general domains (physical, psychological, and social) that can be split into the following 6 subdomains: basic sound perception, advanced sound perception, and speech production (in the physical domain); self-esteem (in the psychological domain); and activity limitations and social interaction (in the social domain). All 60 items in the NCIQ are answerable on a 5-point Likert scale. Before calculating final scores, the items were reversed if applicable and then transformed; therefore, higher scores indicate better HRQoL. Scores from each subdomain were calculated based on the average of these transformed values. A maximum of three missing answers (i.e., not answered or “not applicable”) per subdomain was allowed. Subdomain scores were calculated according to the corrected scoring table published in 2017 [15].

### 2.3. Bias

Data of participating study sites were pooled. The selection criteria set for this study allowed for the enrolment of a homogeneous population within the context of a clinical routine, a real-life setting that allowed a non-biased pooling of the results. All study sites followed the same agreed-upon protocol to prevent a treatment-by-center interaction.

### 2.4. Statistics

The mean with the range (minimum and maximum values) were used to report participant demographic characteristics (e.g., age); the median with the interquartile range (IQR) were used to describe the distribution of the study outcomes; absolute and relative frequencies were used to present qualitative data.

Only participants with pre-operative QoL results (1st visit) and at least one post-activation QoL result were included in the final analyses.

According to the results of the Kolmogorov–Smirnov test and the Shapiro–Wilk test, data were not approximately normally distributed. Hence, non-parametric statistical tests, such as the Wilcoxon signed-rank test for pairwise comparisons and the Friedman test to examine changes over time, were applied.

The Wilcoxon signed-rank test was used to assess the change in HRQoL by comparison of post-activation QoL results with pre-operative QoL results in different CI treatment groups. To adjust for multiple comparisons (Visit 1 to Visit 2 and Visit 1 to Visit 3), the Bonferroni correction had to be applied when interpreting the *p*-values. Hence, a *p*-value of *p* ≤ 0.025 instead of *p* ≤ 0.05 is considered as the significance level.

Statistical analyses were performed with IBM SPSS Statistics Version 25 (IBM, Armonk, NY, USA).

## 3. Results

### 3.1. Participants

Participants were 101 adult unilateral CI users, who were divided into groups based on their preoperative hearing (see Table 1). As stated in the Methods, EAS users from Lassaletta et al. [3] had to be excluded. This accounts for why the present study has 101 and not 111 CI users.

All participants were implanted with a MED-EL (Innsbruck, Austria) CI system (see Table A1 in Appendix A for further details). For further demographic data, please see the CI users (minus the EAS group) in Lassaletta et al. [3].

### 3.2. Generic QoL

A statistically significant increase in HUI-3 score was found in the SSD group between visit 1 and visit 3 (*p* = 0.005) and in the unilateral group between visit 1 and visit 2 (*p* < 0.001) and visit 1 and visit 3 (*p* = 0.001).

**Figure 1:**
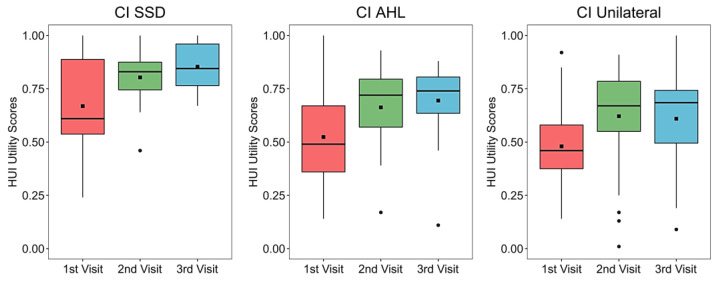
Health Utilities Index (HUI-3) scores for each group at each visit. Mean values are depicted as black squares and the medians as horizontal lines. The black circles represent outliers. Higher scores indicate better quality of life (QoL). CI = cochlear implant, SSD = single-sided deafness, AHL = asymmetric hearing loss, Unilateral = unilateral CI user with bilateral deafness.

A clinically significant improvement (an increase of ≥0.03) between visit 1 and visit 2 and visit 1 and visit 3 was reached in all groups (Figure 1). See Table A2 in Appendix A for median and interquartile scores for each group and at each visit.

### 3.3. Disease-Specific QoL

#### 3.3.1. SSQ12

**Figure 2:**
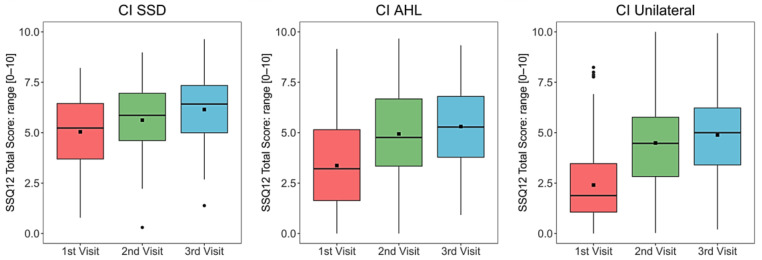
Speech, Spatial, and Qualities of Hearing scale (SSQ12) total scores for each group at each interval. Mean values are depicted as black squares and the medians as horizontal lines. The black circles represent outliers. Higher scores indicate better quality of life (QoL). CI = cochlear implant, SSD = single-sided deafness, AHL = asymmetric hearing loss. Unilateral = unilateral CI user with bilateral deafness.

A statistically significant increase (in total score) was found for all groups at both compared intervals. For the SSD group, significance was *p* = 0.034 between visit 1 and visit 2 and *p* = 0.020 between visit 1 and visit 3. For the AHL and unilateral groups, significance was *p* < 0.001 between both tested intervals (See Figure 2). See Table A3 in Appendix A for median and IQR of total scores for each group at each visit.

#### 3.3.2. NCIQ

For the SSD group, a significant increase was found in basic sound perception between visit 1 and visit 3 (*p* = 0.016) and in activity limitations between visit 1 and visit 3 (*p* = 0.003).

For the AHL group, a significant increase was found in basic sound perception between visit 1 and visit 2 (*p* = 0.005) and visit 1 and visit 3 (*p* = 0.001), in advanced sound perception between visit 1 and visit 2 (*p* = 0.006) and visit 1 and visit 3 (*p* = 0.001), in advanced speech production between visit 1 and visit 2 (*p* = 0.014) and visit 1 and visit 3 (*p* = 0.001), in self-esteem between visit 1 and visit 2 (*p* = 0.016) and visit 1 and visit 3 (*p* = 0.010), and in activity limitations between visit 1 and visit 2 (*p* = 0.009).

**Figure 3:**
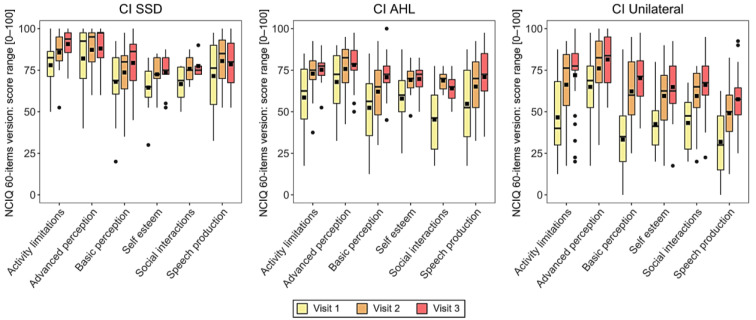
Nijmegen Cochlear Implant Questionnaire (NCIQ) scores for each group at each interval. Mean values are depicted as black squares and the medians as horizontal lines. The black circles represent outliers. Higher scores indicate better quality of life (QoL). CI = cochlear implant, SSD = single-sided deafness, AHL = asymmetric hearing loss, Unilateral = unilateral CI user with bilateral deafness.

For the unilateral group, a significant increase was found in all subcategories between visit 1 and visit 2 and between visit 1 and visit 3. The p value was *p* < 0.001 for all comparisons except advanced sound perception between visit 1 and visit 2 (*p* = 0.008), activity limitations between visit 1 and visit 3 (*p* = 0.001), and social interaction between visit 1 and visit 2 (*p* = 0.013) and visit 1 and visit 3 (*p* = 0.002) (see Figure 3). See Table A4 in Appendix A for the median and IQR of the total scores for each group at each visit.

### 3.4. Audio Processor Daily Use

**Figure 4:**
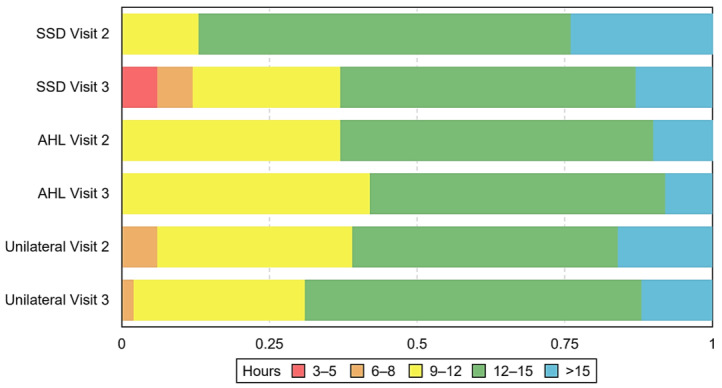
Hours of daily cochlear implant (CI) use for each group at each postoperative interval. Frequency is in hours per day. SSD = single-sided deafness, AHL = asymmetric hearing loss, Unilateral = unilateral CI user with bilateral deafness.

Most participants in each group reported using their CI for ≥12 h per day at both postoperative intervals. At visit 2, 100% of SSD participants, 100% of AHL participants, and 93.9% of unilateral participants used the CI for >9 h per day. At visit 3, this decreased to 87.5% for SSD participants, stayed at 100% for AHL participants, and rose to 97.6% for unilateral participants (see Figure 4). See Table A5 in Appendix A for the percentage of participants in each group who reported using their CI at each range/hours per day.

## 4. Discussion

This study aimed to (1) investigate changes in HRQoL before and after CI provision across different CI indications in a multi-centric/multinational set-up and (2) generate a guidance for clinicians on how HRQoL measures could be used to individualize CI counselling and rehabilitation. The first aim was successful: HRQoL data were collected from a relatively large sample of different types of adult CI users; we hope to achieve the second aim in this section of the paper when we argue that the regular clinical use of both generic and disease-speaking HRQoL assessments should better enable each CI recipient to benefit from individually-tailored rehabilitation and help to generate data that can be used to demonstrate the cost-effectiveness of CI provision, thereby expanding eligibility criteria and reimbursement.

Regarding the HRQoL results, each group’s scores significantly increased upon CI use on the generic measure and at least one of the disease-specific measures. This indicates the beneficial effects of CI use in adult unilateral CI users, regardless of the hearing status of their contralateral ear. These results were expected because it is well known that CI use benefits HRQoL in this population [16,17,18].

Digging deeper into the results, the SSD group had significant improvements on the HUI-3, on the SSQ12 at 12 m but not at 6 m, and only on 2/6 subdomains of the NCIQ at 12 m; in contrast, the unilateral group had significant improvements at all intervals and on all measures. The AHL group had scores similar to the unilateral group, with significant increases on the HUI-3 and SSQ12, but only 5/6 subdomains of the NCIQ at 6 m and 4/6 subdomains at 12 m (see Table 2).

These results indicate that the likelihood of significant HRQoL benefit on particular questionnaires may correspond with the amount of hearing loss in users’ contralateral ear. While it is known that increases in pre- vs. post-implantation speech perception do not necessarily correlate with increases in HRQoL [19,20], this is probably more relevant when comparing the same type of CI users, and not when comparing users with different severities of hearing loss (e.g., a unilateral CI user with bilateral deafness vs. a unilateral CI user with SSD). It stands to reason that a unilateral CI user with bilateral deafness would derive greater HRQoL benefit than a unilateral CI user with SSD; for someone with bilateral deafness, CI use is the difference between functional deafness and the ability to hear, whereas someone with SSD has one ear with normal hearing so they can already participate in the world of sound without a CI, albeit with a diminished ability to understand speech in noise and localize sound [21]. Thus, it may be of future benefit to clinicians to establish if different types of CI users (e.g., with SSD, bilateral CI users, EAS users, etc.) can be expected to score within certain ranges at certain intervals on individual HRQoL questionnaires. Such norm curves could help clinicians establish expectations prior to implantation and, post-implantation, may help troubleshoot during rehabilitation. While such norm curves would necessarily be broad because multiple factors can influence CI users’ HRQoL [22], their existence could still be of clinical value.

The ability to compare a single user’s HRQoL scores against a norm curve also points the way towards HRQoL questionnaires’ ability to enhance opportunities for individualized treatment/rehabilitation. The regular use of HRQoL assessments should allow several benefits. Preoperatively, they could be used to assess and shape patient expectations [23,24]. This could be of real benefit because patients’ preoperative expectations are an important factor in candidacy and assessment [25] and could shape their perception of their postoperative performance [26]. Postoperatively, they could be used as a part of hearing rehabilitation to monitor individual CI users’ progress over time. This could allow for an “individualized rehabilitation approach”, wherein clinicians could use the results to tailor future rehabilitation sessions to each user’s strengths, weaknesses, and goals. A record of results should also serve as a useful reminder for the CI user and/or therapist that progress has been made if it seems to have stalled. We would particularly advocate using disease-specific assessments for this because they appear to be more sensitive than their generic counterparts [3]. Future studies may find it useful to examine if our hypothesis that the regular use of HRQoL questionnaires will lead to more fulfilling rehabilitation is correct.

Nonetheless, generic QoL assessments have a vital role to play. Unlike disease-specific assessments, generic assessments can be used to generate QALYs, which is a generic quantification of disease burden. QALYs are particularly important because they can be used to demonstrate that specific treatments in specific patient groups are a cost-effective use of a health care system’s finite resources. This can be used as a powerful argument for expanding local eligibility criteria and cost coverage, thereby enabling those who might not have received a CI (or a bilateral CI) to benefit from its use. Some recent CI-related publications to this end include Nijmeijer et al. [27], Skarzynski et al. [28], and Cutler et al. [29]. Thus, while generic assessments may be less clinically valuable for individual candidates/users, they are invaluable for enabling future candidates to benefit from CI use. In the present study, we did not use the HUI-3 to generate QALYs because it was outside of our research question. This underscores that while generic QoL assessments can be used to generate QALYs, they do not necessarily always need to be used to that end. Further, future studies, e.g., a review study, could use the data herein for such a purpose.

High rates of daily use should indicate that the user derives benefit from the device. A recent study by Lindquist et al. [30], who used datalogging and a disease-specific measure (the Cochlear Implant Quality of Life (CIQOL)-10 Global measure)), assessed adult CI users with SSD and found that average daily wear time was positively associated with postoperative HRQoL scores; however, data on the relationship between daily device use and HRQoL are limited. While a decline in a user’s daily use time is not necessarily due to reduced benefit, it may alert clinicians that the user is experiencing less satisfaction with device use, which is a cause for intervention.

The present study is not without limitations. Firstly, the follow-up time of 12 months after first fitting, while not unusual in a study on CI recipients/users, is nonetheless still relatively short, especially considering that CI users would be expected to continue using a CI indefinitely. Future studies would benefit from longer follow-up times. Secondly, it assesses only a subset of CI users (adult unilateral CI users of MED-EL devices). Future studies would benefit from including more kinds of CI users, e.g., bilateral CI users, pediatric CI users, etc. Further, while the HUI-3, SSQ12, and NCIQ are commonly used assessments, multiple other assessments exist and could have been used. Regarding which questionnaires to use, clinicians will have their preferences and we refrain from recommending specific questionnaires. As Andries et al. [31] recently pointed out, there is a lack of consensus on which measures are most appropriate, and there is no questionnaire that covers all outcome aspects. Nevertheless, we advocate using questionnaires that are short, validated, and easy-to-use. Regardless of which assessments are used, one must limit the choice of assessments so as to not unduly burden the user/candidate. Cost could also play a role in questionnaire choice: some may find the HUI-3 license prohibitively expensive for everyday clinical use, in which case, using a different generic assessment, e.g., the Assessment of Quality of Life (AQoL)-8D [6], may be preferrable. Lastly, future studies may find it beneficial to use data logging rather than user reporting to collect hours per daily use. Studies on hearing aid users have found that subjective estimates of daily device use are prone to overestimations of up to 2 h per day [32,33].

## 5. Conclusions

Many CI recipients enjoy a significantly increased quality of life (QoL) within the first year of device use. Traditionally, postoperative success was largely based on clinically assessed speech understanding in speech or in noise and, depending on the type of CI user, perhaps sound localization. Increasingly, candidates’/users’ self-assessment of their QoL is being seen as an integral part in showing a fuller, real-life picture of the benefits of CI use. The benefits of preoperative and regular postoperative QoL testing are multiple: results chart an individual user’s progress over time and can be used by clinicians/therapists to identify challenges individual users are facing so to better address them in future sessions. This kind of individualized rehabilitation is probably better conducted with disease-specific measures, of which there are several for people with hearing loss.

The regular use of generic measures could also be of substantial benefit. Generic measures, such as the HUI-3 used herein, can be used to generate QALYs, which can be used to generate the cost-benefit analyses used to demonstrate that CI provision is an efficient use of limited resources. Therefore, in short, the preoperative and regular postoperative use of QoL assessments could potentially enable clinicians/rehabilitationists to better provide personalized care/training and be a key to enabling more people to benefit from CI use. Lastly, regarding QoL assessments, it is important to be judicious in choosing which ones to use. Some assessments are time- and energy-consuming and may not be optimal for all candidates/users.

## Figures and Tables

**Table 1:** Mean and range (minimum and maximum) ages and duration of deafness for each group of participants (SSD = single-sided deafness; AHL = asymmetrical hearing loss; Unilateral = unilateral CI user with bilateral deafness). Note: variations in n are due to missing data.

	n	Age at Time of Testing (Years)	n	Age at Implantation (Years)	n	Duration of Deafness in Implanted Ear (Years)
SSD	17	39.5 (16.4–66.4)	16	39.9 (16.8–66.1)	15	25.3 (9–67)
AHL	26	64.6 (28.3–79.6)	22	64.6 (29.0–78.4)	17	18.8 (2–48)
Unilateral	58	58.7 (24.1–84.7)	52	60.3 (26.2–85.8)	41	20.2 (2–63)

**Table 2:** Did scores increase significantly between those intervals? Significance for each test for each group at each interval. Note: For HUI-3, clinical significance is reported here. For the SSQ12 and NCIQ, results are of statistical significance. CI = cochlear implant, SSD = single-sided deafness, AHL = asymmetric hearing loss, Unilateral = unilateral CI user with bilateral deafness.

	SSD		AHL		Unilateral	
Intervals	1 v 2	1 v 3	1 v 2	1 v 3	1 v 2	1 v 3
HUI-3	Yes	Yes	Yes	Yes	Yes	Yes
SSQ12 (total score)	No	Yes	Yes	Yes	Yes	Yes
NCIQ						
Basic sound perception	No	Yes	Yes	Yes	Yes	Yes
Advanced sound perception	No	No	Yes	Yes	Yes	Yes
Advanced speech production	No	No	Yes	Yes	Yes	Yes
Self-esteem	No	No	Yes	Yes	Yes	Yes
Activity limitations	No	Yes	Yes	No	Yes	Yes
Social interactions	No	No	No	No	Yes	Yes
Total	5/16		13/16		16/16	

## Data Availability

The data presented in this study are available on reasonable request from the corresponding author.

## Appendix A

**Table A1:** Cochlear implant, audio processor, and electrode array provision for each group. SSD = single-sided deafness, AHL = asymmetric hearing loss, Unilateral = unilateral CI user with bilateral deafness.

	SSD	AHL	Unilateral
Cochlear implant			
SONATA^TI^	8	10	15
SYNCHRONY	7	7	6
SYNCHRONY PIN	0	5	15
CONCERTO	1	2	9
CONCERTO PIN	1	1	2
Missing	0	1	11
Total	17	26	58
Audio processor			
SONNET	12	17	39
RONDO	4	2	8
OPUS 2	0	3	5
Missing	1	4	6
Total	17	26	58
Electrode array			
FLEX28	8	13	30
FLEX24	4	6	9
FLEX20	1	2	1
FLEXSOFT	0	1	0
MEDIUM	0	1	0
COMPRESSED	0	1	3
STANDARD	4	1	4
Missing	0	1	11
Total	17	26	58

**Table A2:** HUI-3 median and interquartile range for each group (SSD = single-sided deafness; AHL = asymmetrical hearing loss; Unilateral = unilateral CI user with bilateral deafness) at each visit.

	n	Visit 1	n	Visit 2	n	Visit 3
SSD	16	0.61 (0.54–0.89)	15	0.83 (0.74–0.88)	14	0.85 (0.77–0.96)
AHL	22	0.48 (0.35–0.67)	20	0.68 (0.55–0.79)	19	0.74 (0.65–0.81)
Unilateral	45	0.45 (0.36–0.56)	43	0.67 (0.55–0.79)	40	0.69 (0.50–0.74)

**Table A3:** SSQ12 median and interquartile range of the total scores for each group at each visit are shown. An asterisk * indicates statistical significance (Bonferroni correction: *p* ≤ 0.025). SSD = single-sided deafness; AHL = asymmetrical hearing loss; Unilateral = unilateral CI user with bilateral deafness.

	Visit	n	Total	Wilcoxon Signed-Rank Test (*p*-Values 2-Sided)	
				V1 vs. V2	V1 vs. V3
SSD	1	17	5.1 (4.2–6.4)	0.034	0.020 *
	2	16	6.1 (4.8–6.8)		
	3	16	6.6 (5.8–7.0)		
AHL	1	25	2.8 (1.2–2.5)	<0.001 *	<0.001 *
	2	24	4.5 (3.3–6.5)		
	3	23	5.2 (4.0–6.5)		
Unilateral	1	42	2.3 (1.2–2.5)	<0.001 *	<0.001 *
	2	42	4.4 (2.8–5.6)		
	3	38	4.8 (3.4–6.1)		

**Table A4:** NCIQ median and interquartile range (IQR) of subdomain scores for each group at each visit. An asterisk * indicates statistical significance (Bonferroni correction: *p* ≤ 0.025). SSD = single-sided deafness; AHL = asymmetrical hearing loss; Unilateral = unilateral CI user with bilateral deafness.

		Visit 1		Visit 2		Visit 3		Wilcoxon Signed-Rank Test (p-Values 2-Sided)	
		n	Median (IQR)	n	Median (IQR)	n	Median (IQR)	V1 vs. V2	V1 vs. V3
SSD	Basic sound perception	14	68.8 (60.6–82.5)	11	80.0 (63.8–83.8)	12	86.3 (68.8–90.6)	0.046	0.016 *
	Advanced sound perception	11	92.5 (70.0–97.5)	10	95.0 (80.0–97.5)	9	97.5 (82.5–97.5)	0.159	0.237
	Advanced speech production	10	76.3 (54.4–90.0)	10	85.0 (70.0–93.1)	7	80.0 (67.5–91.3)	0.028	0.237
	Self-esteem	10	65.0 (58.8–74.4)	9	72.5 (70.0–82.5)	9	75.0 (72.5–82.5)	0.107	0.051
	Activity limitations	15	82.5 (71.3–86.3)	14	87.5 (80.6–95.0)	14	93.8 (85.6–97.5)	0.074	0.003 *
	Social interactions	6	68.8 (58.8–76.9)	6	75.0 (69.4–82.5)	5	75.0 (72.5–77.5)	0.042	0.068
AHL	Basic sound perception	18	56.3 (35.6–66.9)	18	65.0 (48.1–75.0)	17	72.5 (67.5–77.5)	0.005 *	0.001 *
	Advanced sound perception	19	72.5 (55.0–83.8)	17	82.5 (67.5–87.5)	18	78.8 (75.0–86.9)	0.006 *	0.001 *
	Advanced speech production	21	52.5 (35.0–75.0)	19	70.0 (52.5–80.0)	19	72.5 (62.5–85.0)	0.014 *	0.001 *
	Self-esteem	19	60.0 (50.0–68.8)	14	70.0 (64.4–74.4)	17	72.5 (65.0–75.0)	0.016 *	0.010 *
	Activity limitations	12	62.5 (45.6–75.6)	12	75.0 (69.4–80.6)	10	77.5 (71.9–79.4)	0.009 *	0.033
	Social interactions	8	46.3 (27.5–60.0)	6	70.0 (63.8–72.5)	8	65.0 (58.1–69.4)	0.068	0.043
Unilateral	Basic sound perception	42	35.0 (20.0–49.4)	39	62.5 (48.8–81.3)	36	71.3 (59.4–80.6)	<0.001 *	<0.001 *
	Advanced sound perception	35	70.0 (52.5–78.8)	36	82.5 (68.8–92.5)	30	83.8 (67.5–95.0)	0.008 *	<0.001 *
	Advanced speech production	40	32.5 (15.0–48.1)	35	50.0 (38.8–61.3)	34	57.5 (48.1–64.4)	<0.001 *	<0.001 *
	Self-esteem	41	42.5 (30.0–52.5)	35	65.0 (45.0–73.8)	35	62.5 (55.0–77.5)	<0.001 *	<0.001 *
	Activity limitations	29	40.0 (30.0–70.0)	27	77.5 (55.0–86.3)	25	77.5 (75.0–85.0)	<0.001 *	0.001 *
	Social interactions	20	47.5 (27.5–55.6)	14	65.0 (52.5–73.1)	17	67.5 (60.0–77.5)	0.013 *	0.002 *

**Table A5:** Percentage of participants from each group who reported using their CI at each range of hours/day. AHL = asymmetrical hearing loss; SSD = single-sided deafness; Unilateral = unilateral CI user with bilateral deafness.

			Hours per Day				
		n	3–5	6–8	9–12	12–15	>15
AHL	Visit 2	19	0%	0%	36.8%	52.6%	10.6%
	Visit 3	24	0%	0%	41.7%	50.0%	8.3%
SSD	Visit 2	16	0%	0%	12.5%	62.5%	25.0%
	Visit 3	16	6.3%	6.3%	25.0%	50.0%	12.5%
Unilateral	Visit 2	49	0%	6.1%	32.7%	44.9%	16.3%
	Visit 3	42	0%	2.4%	28.6%	57.1%	11.9%

## References

[1] Contrera K.J., Betz J., Li L., Blake C.R., Sung Y.K., Choi J.S., Lin F.R. (2016). Quality of life after intervention with a cochlear implant or hearing aid. Laryngoscope.

[2] Thompson N.J., Brown K.D., Dillon M.T. (2022). Cochlear implantation for paediatric and adult cases of unilateral and asymmetric hearing loss. Curr. Opin. Otolaryngol. Head Neck Surg..

[3] Lassaletta L., Calvino M., Sanchez-Cuadrado I., Skarzynski P.H., Cywka K.B., Czajka N., Kutyba J., Tavora-Vieira D., van de Heyning P., Mertens G. (2022). Using Generic and Disease-Specific Measures to Assess Quality of Life before and after 12 Months of Hearing Implant Use: A Prospective, Longitudinal, Multicenter, Observational Clinical Study. Int. J. Environ. Res. Public Health.

[4] Weichbold V., Zelger P., Galvan O., Muigg F. (2023). 5-Year Observation Period of Quality of Life After Cochlear Implantation. Otol. Neurotol..

[5] Horsman J., Furlong W., Feeny D., Torrance G. (2003). The Health Utilities Index (HUI): Concepts, measurement properties and applications. Health Qual. Life Outcomes.

[6] Richardson J., Iezzi A., Khan M.A., Maxwell A. (2014). Validity and reliability of the Assessment of Quality of Life (AQoL)-8D multi-attribute utility instrument. Patient.

[7] Dixon P.R., Shapiro J., Tomlinson G., Cottrell J., Lui J.T., Falk L., Chen J.M. (2023). Health State Utility Values Associated with Cochlear Implants in Adults: A Systematic Review and Network Meta-Analysis. Ear Hear..

[8] McRackan T.R., Hand B.N., Velozo C.A., Dubno J.R. (2021). Validity and reliability of the Cochlear Implant Quality of Life (CIQOL)-35 Profile and CIQOL-10 Global instruments in comparison to legacy instruments. Ear Hear..

[9] Andries E., Gilles A., Topsakal V., Vanderveken O.M., Van de Heyning P., Van Rompaey V., Mertens G. (2021). Systematic Review of Quality of Life Assessments after Cochlear Implantation in Older Adults. Audiol. Neurootol..

[10] Noble W., Jensen N.S., Naylor G., Bhullar N., Akeroyd M.A. (2013). A short form of the Speech, Spatial and Qualities of Hearing scale suitable for clinical use: The SSQ12. Int. J. Audiol..

[11] Hinderink J.B., Krabbe P.F., Van Den Broek P. (2000). Development and application of a health-related quality-of-life instrument for adults with cochlear implants: The Nijmegen cochlear implant questionnaire. Otolaryngol. Head Neck Surg..

[12] Cox R.M., Alexander G.C. (1995). The abbreviated profile of hearing aid benefit. Ear Hear..

[13] Kompis M., Pfiffner F., Krebs M., Caversaccio M.D. (2011). Factors influencing the decision for Baha in unilateral deafness: The Bern benefit in single-sided deafness questionnaire. Adv. Otorhinolaryngol..

[14] Summerfield A.Q., Kitterick P.T., Goman A.M. (2022). Development and Critical Evaluation of a Condition-Specific Preference-Based Measure Sensitive to Binaural Hearing in Adults: The York Binaural Hearing-Related Quality-of-Life System. Ear Hear..

[15] (2017). Corrigendum. Otolaryngol. Head Neck Surg..

[16] Peters J.P.M., van Heteren J.A.A., Wendrich A.W., van Zanten G.A., Grolman W., Stokroos R.J., Smit A.L. (2021). Short-term outcomes of cochlear implantation for single-sided deafness compared to bone conduction devices and contralateral routing of sound hearing aids-Results of a Randomised controlled trial (CINGLE-trial). PLoS ONE.

[17] Borre E.D., Kaalund K., Frisco N., Zhang G., Ayer A., Kelly-Hedrick M., Reed S.D., Emmett S.D., Francis H., Tucci D.L. (2023). The Impact of Hearing Loss and Its Treatment on Health-Related Quality of Life Utility: A Systematic Review with Meta-analysis. J. Gen. Intern. Med..

[18] Dillon M.T., Kocharyan A., Daher G.S., Carlson M.L., Shapiro W.H., Snapp H.A., Firszt J.B. (2022). American Cochlear Implant Alliance Task Force Guidelines for Clinical Assessment and Management of Adult Cochlear Implantation for Single-Sided Deafness. Ear Hear..

[19] Capretta N.R., Moberly A.C. (2016). Does quality of life depend on speech recognition performance for adult cochlear implant users?. Laryngoscope.

[20] McRackan T.R., Hand B.N., Velozo C.A., Dubno J.R. (2019). Association of Demographic and Hearing-Related Factors with Cochlear Implant-Related Quality of Life. JAMA Otolaryngol. Head Neck Surg..

[21] Galvin J.J., Fu Q.J., Wilkinson E.P., Mills D., Hagan S.C., Lupo J.E., Padilla M., Shannon R.V. (2019). Benefits of Cochlear Implantation for Single-Sided Deafness: Data from the House Clinic-University of Southern California-University of California, Los Angeles Clinical Trial. Ear Hear..

[22] Skidmore J.A., Vasil K.J., He S., Moberly A.C. (2020). Explaining Speech Recognition and Quality of Life Outcomes in Adult Cochlear Implant Users: Complementary Contributions of Demographic, Sensory, and Cognitive Factors. Otol. Neurotol..

[23] McRackan T.R., Hand B.N., Chidarala S., Dubno J.R. (2022). Understanding Patient Expectations Before Implantation Using the Cochlear Implant Quality of Life-Expectations Instrument. JAMA Otolaryngol. Head Neck Surg..

[24] Illg A., Bräcker T., Batsoulis C., Opie J.M., Lesinski-Schiedat A. (2022). CI decision making and expectations by older adults. Cochlear Implant. Int..

[25] Prentiss S., Snapp H., Zwolan T. (2020). Audiology Practices in the Preoperative Evaluation and Management of Adult Cochlear Implant Candidates. JAMA Otolaryngol. Head Neck Surg..

[26] Harris M.S., Capretta N.R., Henning S.C., Feeney L., Pitt M.A., Moberly A.C. (2016). Postoperative Rehabilitation Strategies Used by Adults with Cochlear Implants: A Pilot Study. Laryngoscope Investig. Otolaryngol..

[27] Nijmeijer H.G.B., Groenewoud H.M.M., Mylanus E.A.M., Goedegebure A., Huinck W.J., van der Wilt G.J. (2023). Impact of Expanding Eligibility Criteria for Cochlear Implantation Dynamic Modeling Study. Laryngoscope.

[28] Skarzynski P.H., Ciesla K., Lorens A., Wojcik J., Skarzynski H. (2021). Cost-Utility Analysis of Bilateral Cochlear Implantation in Adults with Severe to Profound Sensorineural Hearing Loss in Poland. Otol. Neurotol..

[29] Cutler H., Gumbie M., Olin E., Parkinson B., Bowman R., Quadri H., Mann T. (2022). The cost-effectiveness of unilateral cochlear implants in UK adults. Eur. J. Health Econ..

[30] Lindquist N.R., Holder J.T., Patro A., Cass N.D., Tawfik K.O., O’Malley M.R., Bennett M.L., Haynes D.S., Gifford R.H., Perkins E.L. (2023). Cochlear Implants for Single-Sided Deafness: Quality of Life, Daily Usage, and Duration of Deafness. Laryngoscope.

[31] Andries E., Lorens A., Skarżyński P.H., Skarzynski H., Calvino M., Gavilan J., Lassaletta L., Tavora-Vieira D., Acharya A., Kurz A. (2022). Evaluating the Revised Work Rehabilitation Questionnaire in Cochlear Implant Users Cochlear Implant Outcome Assessment Based on the International Classification of Functioning, Disability, and Health (ICF). Otol. Neurotol..

[32] Walker E.A., Spratford M., Moeller M.P., Oleson J., Ou H., Roush P., Jacobs S. (2013). Predictors of hearing aid use time in children with mild-to-severe hearing loss. Lang. Speech Hear. Serv. Sch..

[33] Walker E.A., McCreery R.W., Spratford M., Oleson J.J., Van Buren J., Bentler R., Roush P., Moeller M.P. (2015). Trends and Predictors of Longitudinal Hearing Aid Use for Children Who Are Hard of Hearing. Ear Hear..

